# Radiological identification of neuromyelitis optica in a patient presented with unexplained weight loss and generalized weakness: a case report and literature review

**DOI:** 10.1097/MS9.0000000000000887

**Published:** 2023-05-24

**Authors:** Oadi N. Shrateh, Afnan W.M. Jobran, Zaina Khaled, Momen A. Zaid, Ahmad A. Dallashi

**Affiliations:** a Faculty of Medicine, Al-Quds University, Jerusalem; b Department of Internal Medicine, Palestinian Medical Complex, Ramallah, Palestine

**Keywords:** aquaporin-4 antibodies, case report, MRI, neuromyelitis optica

## Abstract

Central nervous system (CNS) inflammatory demyelinating disease known as neuromyelitis optica spectrum disorder (NMOSD) is characterized by recurrent inflammatory events that primarily affect the optic nerves and spinal cord; it may also affect the hypothalamus, area postrema, and periaqueductal gray matter. The NMOSD-specific aquaporin-4 antibody (AQP4-IgG) is available. Myelin oligodendrocyte glycoprotein antibodies (MOG-IgG) have recently been discovered in a group of patients who do not have AQP4-IgG.

**Case presentation:**

A 29-year-old female presented to the hospital with the complaint of blurry vision in her right eye and left eye ptosis for 2 days. Two months ago, the patient had a history of generalized fatigue with continuous documented fever with an average of 38.5°C, which was relieved by acetaminophen and ibuprofen. She also complained of continuous hiccups that increased at night and interfered with her sleep pattern and breathing; they lasted for 3 weeks and disappeared suddenly. She had also developed episodes of vomiting and could not tolerate food intake due to which she lost 6 kg within 3 weeks. She was later diagnosed with neuromyelitis optica (NMO) using radiological neuroimaging.

**Clinical discussion:**

Early and correct diagnosis, followed by urgent treatment for acute exacerbations and the prevention of further relapses, are essential for treating NMO spectrum illnesses since they entail significant morbidity and, occasionally, fatality.

**Conclusion:**

The patient mentioned here represents a typical example of NMO disease. This case emphasizes the presence of this disease in our environment and the importance of accurately diagnosing this ailment, even in a context with minimal resources, to prevent disability.

## Introduction

Highlights

- Neuromyelitis optica spectrum disorder (NMOSD) affects the central nervous system (CNS), frequently targeting the spinal cord or optic nerves.
- We affirm the significance of neuroimaging including magnetic resonance imaging in the diagnosis of NMOSD even if the clinical and/or laboratory findings are not directed toward the correct diagnosis.

Since the myelin oligodendrocyte glycoprotein (MOG) is expressed on the outside of the myelin sheath, autoantibodies are likely to attack it^1^. It has been acknowledged that a multiple sclerosis (MS) subtype with the clinical phenomenology of optic neuromyelitis is a separate entity^2^. In 2015, the idea of neuromyelitis optica spectrum disorder (NMOSD) was put forth by the updated international diagnostic standard criteria^3^. Individuals were then identified as having NMOSD if they tested positive for anti-aquaporin-4 (AQP4) antibodies or displayed one of the key clinical symptoms (including optic neuritis (ON), myelitis, and brain disorders)^3^. ON with positivity to an anti-MOG antibody and negativity to an anti-AQP4 antibody may occasionally be a disease concept that also satisfies the NMOSD criteria if it exhibits one of the key clinical symptoms. On the other hand, in a few instances where NMOSD criteria have not been met, ON is thought to be brought on by a condition called MOGAD (anti-MOG antibody-associated illness), which is unrelated to neuromyelitis optica (NMO) or MS. It is interesting to note that myeloperoxidase antineutrophil cytoplasmic antibodies (MPO-ANCA) positive has only been documented in a small percentage of NMO patients^4^. This case report has been reported in line with the SCARE 2020 Criteria^5^.

## Case presentation

S.H., a 29-year-old Palestinian female nurse, presented to our hospital with the complaint of blurry vision in her right eye and left eye ptosis for 2 days. Two months ago, the patient had a history of generalized fatigue with continuous documented fever with an average of 38.5°C, which was relieved by acetaminophen and ibuprofen. She also complained of continuous hiccups that increased at night and interfered with her sleep pattern and breathing; they lasted for 3 weeks and disappeared suddenly. She had also developed episodes of vomiting and could not tolerate food intake due to which she lost 6 kg within 3 weeks. Bowel and bladder habits were normal except for 1 month’s history of constipation. There was no history of seizures or vertigo. She had no known addiction history. The patient reported no personal and/or family history of cancer, any acute, repeat, or discontinued medications, any allergies, or any genetic or psychosocial issues. Past medical and surgical histories were free.

Upon admission, the physical assessment was done and the patient looked reasonable and afebrile with normal vital signs (temperature 36°C, O_2_ saturation 96% on room air, blood pressure 117/77 supine, right arm). Central nervous system (CNS) examination showed normal Glasgow Coma Scale (GCS) of E4V5M6; motor examination showed normal power (5/5), normal tones, and normal reflexes (+2) in all of her limbs; cranial nerve function tests were intact; sensory examination revealed normal findings at all levels: cerebral signs were negative; the patient can stand but sways more when her eyes are opened; there is no widened gait or limping when walking; walking one foot in front of the other was unsuccessful.

Furthermore, the ocular examination was performed and showed left eye ptosis, blurry vision in her right eye, normal ocular movements in all cardinal directions of gaze, no diplopia, pupils were normal in size and reactive, the visual field was intact in both eyes, and the face examination was unremarkable. Cardiovascular, respiratory, and gastrointestinal systems were normal except for mild epigastric tenderness, erythema of the pharynx, and pus over the right tonsil with obvious enlargement.

**Figure 1:**
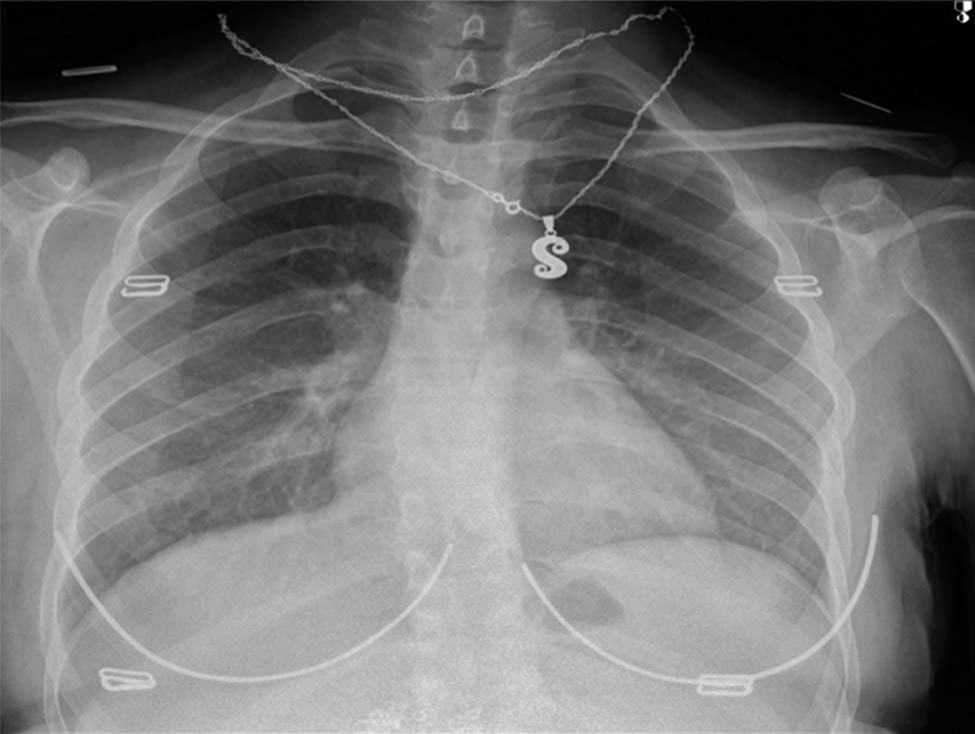
Normal erect posteroanterior (PA) chest X-ray.

Systemic investigations revealed microcytic anemia with a low hemoglobin level of 10.6 g/dl, hematocrit of 33.3%, mean corpuscular volume (MCV) of 69.8 fl, and Mean corpuscular hemoglobin of 22.1 pg; her erythrocyte sedimentation rate (ESR) was 60 mm/h and C-reactive protein (CRP) was 7.6; she had normal renal function tests (RFT) and liver function tests (LFT), normal blood sugar, thyroid hormone assay, urinalysis, and chest X-ray (Fig. 1).

Further review of the cerebrospinal fluid (CSF) showed slightly elevated glucose levels and negative oligoclonal banding with no other significant findings. Antinuclear antibody (ANA) was positive, whereas double-stranded DNA (dsDNA), Sjogren’s syndrome-related antigen A (SSA) (anti-Ro) antibody, and Scl 70 were negative.

The patient was advised to undergo serum anti-AQP4 antibody testing and anti-MOG antibody testing, where both showed a negative result. While her NMO antibodies were positive. The patient was started on oral steroids and is currently under close follow-up with an internist, ophthalmologist, and rheumatologist for her case.

**Figure 2:**
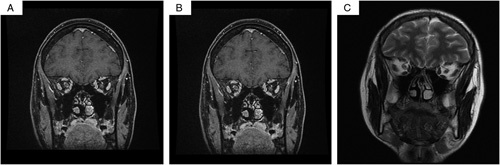
(A) MRI of the brain with a coronal section with no contrast done on 1.5 T showed enhanced swollen left optic nerve with high T2-signal and perioptic fat stranding compared to the normal left side, suggestive of optic neuritis. (B) MRI with a coronal section of the brain with no contrast done on 1.5 T showed enhanced swollen left optic nerve with high T2-signal and perioptic fat stranding compared to the normal left side, suggestive of optic neuritis. (C) MRI of the brain with a coronal section with no contrast done on 1.5 T showed enhanced swollen left optic nerve with high T2-signal and perioptic fat stranding compared to the normal left side, suggestive of optic neuritis.

**Figure 3:**
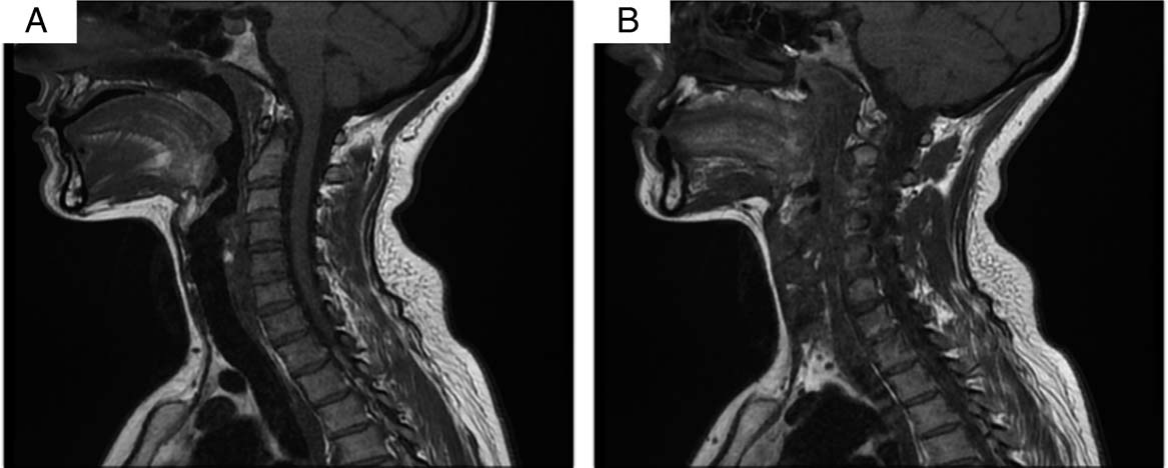
(A) Multiplanar multisequence MRI of the cervical spine with contrast done on 1.5 T showed normal cervical curvature with no significant spinal cord stenosis or neural foramina narrowing. (B) Multiplanar multisequence MRI of the cervical spine with contrast done on 1.5 T showed intact prevertebral and paravertebral soft tissue.

A multiplanar multisequence magnetic resonance imaging (MRI) of the brain with no contrast was done on 1.5 T and showed an enhanced swollen left optic nerve with high T2-signal and preoptic fat stranding compared to the normal left side, suggestive of ON (Fig. 2A–C). MRI spine with contrast showed normal cervical curvature with no significant spinal cord stenosis or neural foramina narrowing and intact prevertebral and paravertebral soft tissue (Fig. 3A, B). The patient was followed up for 6 months with significant improvement. The patient also had a good tolerance for pharmacological agents without any reported complications or adverse events.

## Discussion

An inflammatory condition known as NMOSD affects the CNS, frequently targeting the spinal cord or optic nerves. Although it was formerly believed to be closely connected to MS, more recent research has shown that it represents a distinct clinical and pathophysiologic entity^5^. Devic’s syndrome or Devic’s sickness are other names for this ailment. Recurrent longitudinally extensive myelitis (greater than a three-vertebral segment spinal cord lesion observed on MRI) and recurrent ON are two NMO spectrum illnesses^3^.

Finding antibodies to aquaporin-4, a water channel on astrocytic foot processes that are highly expressed in the spinal cord, brainstem, and optic nerves^5^. It is regarded as an extra criterion supporting the diagnosis and is a highly specific biomarker for NMO as well as for NMO spectrum illnesses^3^. The consensus diagnostic criteria were modified by the International Panel for Neuromyelitis Optica Diagnosis (IPND) in 2015 to reflect pertinent advancements^3^. Given that she satisfies this diagnostic requirement, I am reporting this case.

Early and correct diagnosis, followed by urgent treatment for acute exacerbations and the prevention of further relapses, are essential for treating NMO spectrum illnesses since they entail significant morbidity and, occasionally, fatality^5^. At a median age of 39 years, NMO is up to nine times more common in women. NMO, as opposed to MS, affects non-White people more frequently^6^. East Africa has only a few cases of NMO documented^7,8^. Two cases have been documented in Ethiopia; however, only one has been previously published^8^.

Visual, motor, sensory, and constitutional problems are the most common NMOSD-presenting symptoms (such as vomiting, fever, and seizures). In the majority of pediatric studies of NMOSD, Transverse myelitis or ON occurred as the first clinical event in 30–50% of patients and ON or both in 50–75% of children^9^. Ataxia, encephalopathy, and cranial nerve dysfunction are among the symptoms that can manifest as ophthalmoparesis or region postrema syndrome^10^. Optic nerve inflammation, often known as ON, is characterized by a unilateral or bilateral decrease of visual acuity along with pain when moving the eyes. In NMOSD, ON is frequently bilateral, longitudinally widespread, and prefers posterior parts of the optic nerve, especially the optic chiasm^11^.

Treatment for NMOSD includes both preventive measures and the management of acute attacks. Since relapses can result in lifelong disability, all patients with suspected NMOSD should get treatment during the acute period^12^. Given the lack of controlled research, treatment for children should be given based on what has worked for adults. One typical method of treatment starts with high-dose intravenous methylprednisolone (IVMP). For 5 days straight, a dose of 20 mg/kg/day up to 1000 mg is advised. After that, a gradual drop in oral glucocorticoids is advised, followed by long-term maintenance^13^. Plasma exchange (PLEX) should be taken into consideration if the initial reaction to steroids is insufficient^14^. Five exchanges spread out over 5–10 days make up PLEX therapy^15^. Abboud *et al.* discovered in their study that PLEX+IVMP was linked to better outcomes than IVMP alone, particularly in individuals receiving preventive therapy^16^. If PLEX is not an option due to contraindications, 2 g/kg of intravenous immunoglobulin (IVIG) is advised^17^. However, the effectiveness of IVIG is debatable as only a small number of studies have examined its impact on acute exacerbations of NMOSD. In the acute phase, all of the patients in our sample received IVMP treatment; only two also needed PLEX therapy^18^.

## Conclusion

In this clinical report, we affirm the significance of neuroimaging, including MRI, in the diagnosis of NMOSD, even if the clinical and/or laboratory findings are not directed toward the correct diagnosis. This case emphasizes the presence of this disease in our environment and the importance of accurately diagnosing this ailment, even in a context with minimal resources, to prevent disability.

## Ethical approval

Our institution has exempted this study from ethical review.

## Consent

Written informed consent was obtained from the patient for the publication of this case report. A copy of the informed consent is available for review by the Editor-in-Chief of this journal on request.

## Sources of funding

The authors declare that writing and publishing this manuscript was not funded by any organization.

## Author contribution

O.N.S., A.W.M.J., and Z.K.: writing the manuscript; Z.K. and M.A.Z.: imaging description; O.N.S. and A.A.D.: reviewing and editing the manuscript.

## Conflicts of interest disclosure

The authors declare that there are no conflicts of interest regarding the publication of this article.

## Research registration unique identifying number (UIN)

1. Name of the registry: not applicable.
2. Unique identifying number or registration ID: not applicable.
3. Hyperlink to your specific registration (must be publicly accessible and will be checked): not applicable.

## Guarantor

Oadi N. Shrateh.

## Data availability statement

The dataset is available upon reasonable request.

## Provenance and peer review

Not commissioned, externally peer-reviewed.

## Authorship

All authors attest that they meet the current ICMJE criteria for authorship.
